# Sinapic acid alleviates renal ischemia-reperfusion injury by regulating oxidative stress, apoptosis, and inflammation

**DOI:** 10.55730/1300-0144.6052

**Published:** 2025-06-18

**Authors:** Mustafa Can GÜLER, Fazile Nur EKİNCİ AKDEMİR, Ersen ERASLAN, Ayhan TANYELİ, Derya GÜZEL ERDOĞAN, Behzat TEBRİZİ

**Affiliations:** 1 Department of Physiology, Faculty of Medicine, Atatürk University, Erzurum, Turkiye; 2 Department of Physiology, Erzurum Medicine Faculty, Health Sciences University, Erzurum, Turkiye; 3 Department of Physiology, Faculty of Medicine, Bandırma Onyedi Eylül University, Balıkesir, Turkiye; 4 Department of Physiology, Faculty of Medicine, Sakarya University, Sakarya, Turkiye; 5 Department of Pathology, Faculty of Veterinary Medicine, Dicle University, Diyarbakır, Turkiye

**Keywords:** Sinapic acid, renal ischemia-reperfusion, oxidative stress, inflammation, apoptosis

## Abstract

**Background/aim:**

Acute kidney injury (AKI) is a major clinical issue, frequently resulting from ischemia-reperfusion (I/R) injury. Sinapic acid (SA), a natural phenolic molecule included in numerous plant-based foods, exhibits antiapoptotic, antioxidant, and antiinflammatory properties. This study aimed to investigate the renoprotective effects of SA in an I/R-induced acute kidney injury (AKI) model.

**Materials and methods:**

Sprague–Dawley male rats (n = 32) were randomly assigned to four groups: sham, I/R, SA 20 mg/kg, and SA 40 mg/kg. SA was administered intraperitoneally before reperfusion. Renal tissues were examined using biochemical, histopathological, and immunohistochemical methods, focusing on oxidative stress, cytokine expression, and apoptosis markers.

**Results:**

I/R induced significant oxidative stress, elevated proinflammatory cytokines, and tubular damage. Treatment with SA, particularly at 40 mg/kg, significantly improved antioxidant defenses, reduced inflammatory cytokine levels, and attenuated tubular necrosis and apoptosis, as confirmed by decreased caspase-3 and HAVCR1 (also known as KIM-1) expression.

**Conclusion:**

SA significantly ameliorated renal I/R injury by modulating apoptosis, inflammation, and oxidative stress. These findings support the therapeutic efficacy of SA in AKI and highlight the need for further translational research.

## Graphical Abstract

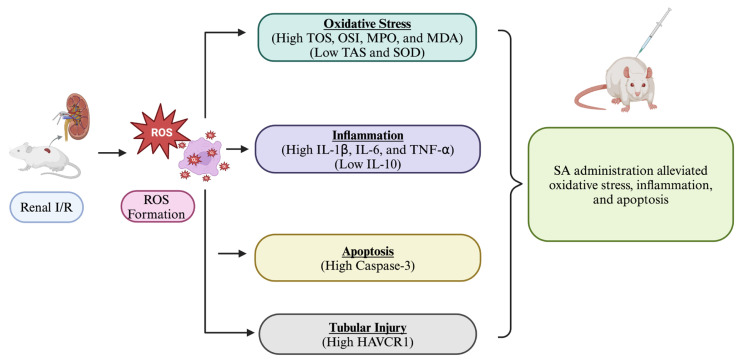

## 1. Introduction

Acute kidney injury (AKI) is a common and serious medical condition characterized by a rapid decline in renal function. It is associated with high mortality and morbidity if not promptly treated [1, 2]. AKI generally occurs as a result of ischemia-reperfusion (I/R) injury, particularly in surgical, transplant, and critical care settings [3]. The pathogenesis of I/R-induced AKI involves complex molecular and cellular processes (apoptosis, inflammation, oxidative stress, etc.), which together contribute to irreversible renal tissue injury [4, 5]. During reperfusion, the rapid influx of oxygen and nutrients triggers excessive generation of reactive oxygen species (ROS), disrupting redox homeostasis and causing damage to lipids, nucleic acids, and proteins [6, 7]. Malondialdehyde (MDA) and myeloperoxidase (MPO) are recognized indicators of lipid peroxidation and oxidative injury. On the other hand, superoxide dismutase (SOD) and total antioxidant status (TAS) reflect the endogenous antioxidant defense capacity [8].

In addition to oxidative stress, renal I/R triggers an acute inflammatory process defined by proinflammatory cytokine upregulation, such as interleukin-6 (IL-6), tumor necrosis factor-alpha (TNF-α), and interleukin-1 beta (IL-1β), which further aggravate renal injury [9–11]. The expression of kidney injury molecule-1 (HAVCR1, also known as KIM-1), a well-established biomarker of tubular damage, is also significantly elevated following I/R insult and correlates with epithelial injury and inflammation

[12, 13]. Apoptosis is another critical process in I/R-related AKI, with caspase-3 acting as a key executioner in renal tubular epithelial apoptosis [14, 15].

Sinapic acid (SA) is a hydroxycinnamic acid derivative abundantly found in cereals, vegetables, fruits, mustard seed, rapeseed, and various spices [16–18]. It has strong antimicrobial, antiinflammatory, and antioxidant effects [19]. SA has been examined in several experimental models of organ damage, including myocardial I/R injury [20], gentamicin-induced nephrotoxicity [21, 22], and testicular injury [23]. However, the therapeutic role of SA in renal I/R injury remains insufficiently explored.

Considering the multifactorial nature of renal I/R injury and the pharmacological potential of SA, this study aimed to evaluate the protective effects of SA on oxidative stress, inflammation, and apoptosis in a renal I/R-induced AKI rat model. While previous studies have demonstrated the nephroprotective effects of SA in models such as gentamicin- and cisplatin-induced nephrotoxicity, its role in renal I/R injury—a clinically relevant cause of AKI—remains insufficiently explored. Thus, this study addresses a significant gap by assessing the therapeutic potential of SA in this context. We hypothesized that SA administration before reperfusion would attenuate kidney injury by modulating these interrelated pathological mechanisms.

## 2. Materials and methods

### 2.1. Chemical agents

**Figure 1:**
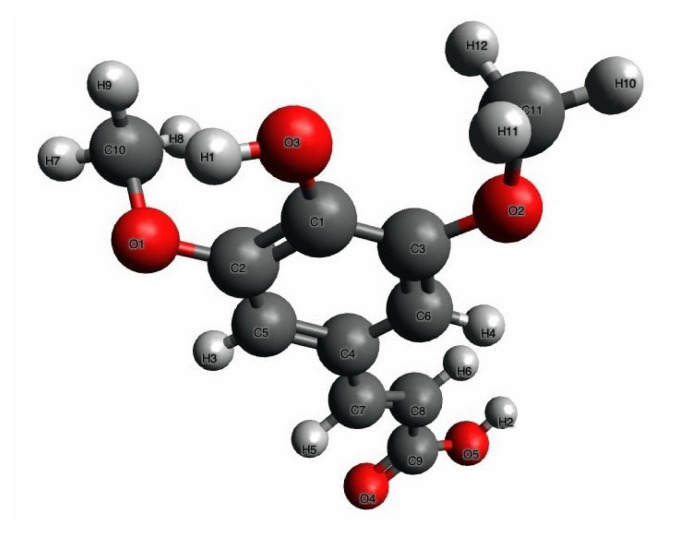
The 3D chemical structure of sinapic acid (created with Avogadro version 1.2.0, http://avogadro.cc/).

Surgical disinfection was carried out using a 10% povidone-iodine solution (Batticon; Adeka, Samsun, Türkiye). Anesthesia was achieved via intraperitoneal (i.p.) administration of ketamine (Ketalar; Pfizer, İstanbul, Türkiye) with xylazine hydrochloride (Rompun, Bayer, İstanbul, Türkiye). Thiopental sodium (CAS no: 71–73–8; İbrahim Ethem Ulagay, İstanbul, Türkiye) was employed for euthanasia. Sinapic acid (SA; ≥ 98% purity, CAS no: 530–59–6) was purchased from Sigma-Aldrich (Steinheim, Germany) (Figure 1).

### 2.2. Ethical approval

The Animal Experiments Local Ethics Committee of Atatürk University approved the study procedure (Approval date: 26 July 2018, Protocol no: 159). All experimental procedures were conducted in full compliance with the ARRIVE guidelines (http://arriveguidelines.org).

### 2.3. Experimental animals

Thirty-two healthy Sprague–Dawley male rats (200–250 g, 12–16 weeks old) were acquired from Atatürk University Experimental Animal Research and Application Center. Rats were kept in polypropylene cages within conventional laboratory conditions (12 h light/dark cycle, 22 ± 2 °C, 55% humidity), with free access to water and a regular diet. Food was withdrawn 24 h before surgery.

### 2.4. Preoperative preparation

Animals were anesthetized and placed in a dorsal recumbent position. The surgical region was shaved and then disinfected with 10% povidone-iodine. Anesthesia was maintained with a ketamine/xylazine combination, as described previously [24].

### 2.5. Experimental design and renal I/R model

The rats were split into four equal groups at random (n = 8):

Sham group: Underwent laparotomy and closure without vascular clamping.

I/R group: Following an incision, bilateral renal artery occlusion was performed using steel vascular clamps for 60 min, followed by 24 h reperfusion [25].

SA 20 mg/kg group: Received 20 mg/kg SA via i.p. injection approximately 30 min before reperfusion, followed by the I/R protocol. SA doses were based on previous studies [26].

SA 40 mg/kg group: Received 40 mg/kg SA administered at the same time point, ensuring consistency in administration and facilitating comparative dose-response analysis. SA doses were based on previous studies [26].

Following reperfusion, animals were euthanized by isoflurane and thiopental sodium [27]. Kidneys were excised and processed for biochemical, histopathological, and immunohistochemical analysis.

### 2.6. Biochemical analysis

Renal tissue levels of myeloperoxidase (MPO) activity, superoxide dismutase (SOD) levels, and malondialdehyde (MDA) values were determined using previously described methods [28–30], with commercial kits from Thermo Fisher Scientific (Waltham, MA, USA). Total oxidant status (TOS) and TAS levels were assessed via Rel Assay Diagnostics kits (Gaziantep, Türkiye), and oxidative stress index (OSI) was calculated as the TOS/TAS ratio [31]. IL-6, IL-10, IL-1β, and TNF-α levels were measured using ELISA kits from Elabscience (Wuhan, China) with the following catalog numbers: IL-6 (E-EL-R0015), IL-1β (E-EL-R0012), TNF-α (E-EL-R0019), and IL-10 (E-EL-R0016).

### 2.7. Histopathological evaluation

Renal tissue sections were sliced to a thickness of 5 μm and subsequently stained with hematoxylin and eosin (H & E) after they were fixed in 10% buffered formalin and placed in paraffin. A light microscope was used to inspect the sections (Olympus BX51, Olympus, Tokyo, Japan; camera: Taptek, İstanbul, Türkiye) for tubular necrosis, hemorrhage, and degeneration. Lesions were scored as none (−), mild (+), moderate (++), or severe (+++) [32].

### 2.8. Immunohistochemical examination

Paraffin-embedded sections were subjected to standard antigen retrieval with citrate buffer (pH 6.0) and microwave heating. The use of 3% hydrogen peroxide inhibited endogenous peroxidase activity. Sections were exposed to primary antibodies for incubation: cleaved caspase-3 (Novus Biologicals, Cettennial, CO, USA; Cat. no: NB600–1235, 1:100) and HAVCR1 (Bioss, Woburn, MA, USA; Cat. no: bs–2713R, 1:200). A mouse/rabbit HRP-DAB detection kit was used for visualization. Immunopositivity was scored semiquantitatively as none (−), mild (+), moderate (++), or severe (+++) [8].

### 2.9. Statistical analysis

Biochemical data analysis was conducted using SPSS version 20.0 (IBM Corp., Armonk, NY, USA), with the Shapiro–Wilk test applied to assess data normality. Following confirmation of normal distribution, the homogeneity of variances was evaluated to ensure the validity of parametric tests. A one-way analysis of variance (ANOVA) was used, then Tukey’s honest significant difference (HSD) test was preferred for post hoc comparisons. A significance level of p < 0.05 was used, and data are presented as mean ± standard error of the mean (SEM).

For histopathological and immunohistochemical evaluations, SPSS version 20.0 was used. The Kruskal–Wallis test, a nonparametric method, was used to identify differences among groups. In cases where significant differences were observed, pairwise comparisons were conducted using the Mann–Whitney U test. Duncan’s test was applied to compare group means when applicable, and a p-value below 0.05 was considered statistically significant.

## 3. Results

### 3.1. Biochemical results

**Figure 2:**
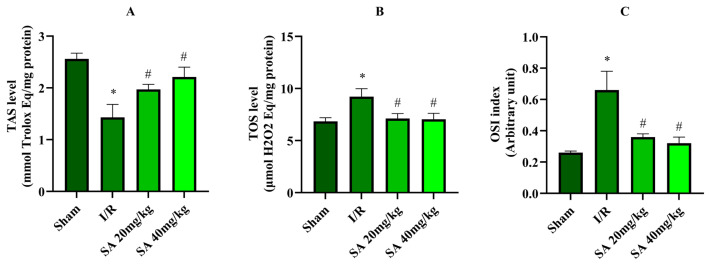
Levels of oxidative stress and antioxidative status parameters across experimental groups. A) TAS (mmol Trolox Eq/mg), B) TOS (μmol H_2_O_2_ Eq/mg), and C) OSI (arbitrary unit) in the sham, I/R, SA 20 mg/kg, and SA 40 mg/kg groups. Data are presented as mean ± standard error of the mean (SEM). *p = 0.026–0.037 compared to the sham group; #p = 0.025–0.033 compared to the I/R group (one-way ANOVA with Tukey’s HSD test).

Figure 2 illustrates TAS, TOS, and OSI levels across the experimental groups. The I/R group showed a reduction in TAS compared to the sham group (Figure 2A, p = 0.026), indicating compromised antioxidant defenses. SA treatment significantly increased TAS at both 20 mg/kg (p = 0.031) and 40 mg/kg (p = 0.029) doses relative to the I/R group, with greater improvement observed at 40 mg/kg.

TOS levels were elevated in the I/R group compared to the sham (p < 0.044), reflecting increased oxidative burden (Figure 2B). SA administration significantly reduced TOS at both 20 mg/kg (p = 0.038) and 40 mg/kg (p = 0.025) doses, approaching levels observed in the sham group. Consequently, the OSI (TOS/TAS ratio) was higher in the I/R group (p < 0.037, Figure 2C) and diminished by both SA 20 mg/kg (p = 0.033) and 40 mg/kg (p = 0.027) doses, indicating restoration of redox balance.

**Figure 3:**
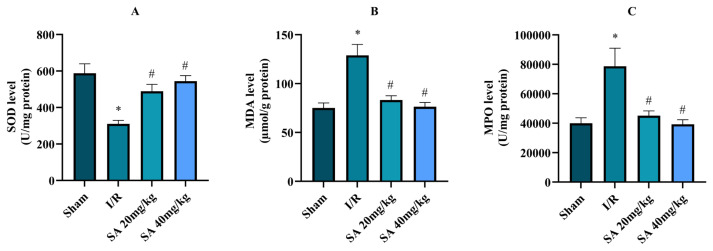
Levels of oxidative stress and antioxidative status parameters across experimental groups. A) SOD activity (U/mg protein), B) MDA (μmol/mg protein), and C) MPO activity (U/mg protein) in the sham, I/R, SA 20 mg/kg, and SA 40 mg/kg groups. Data are presented as mean ± standard error of the mean (SEM). *p = 0.020–0.033 compared to the sham group; #p = 0.019–0.028 compared to the I/R group (one-way ANOVA with Tukey’s HSD test).

As shown in Figure 3, SOD activity declined in the I/R group relative to sham (p < 0.033, Figure 3A), suggesting impaired antioxidant capacity. SA treatment at 20 mg/kg (p = 0.029) and 40 mg/kg (p = 0.023) increased SOD levels compared to the I/R group. MDA levels, representing lipid peroxidation, were significantly elevated in the I/R group (p < 0.020, Figure 3B) and were reduced by both 20 mg/kg (p = 0.025) and 40 mg/kg (p = 0.021) SA treatment. Similarly, MPO activity was elevated in the I/R group (p < 0.022, Figure 3C), and SA reduced MPO levels at both 20 mg/kg (p = 0.028) and 40 mg/kg (p = 0.019) doses.

**Figure 4:**
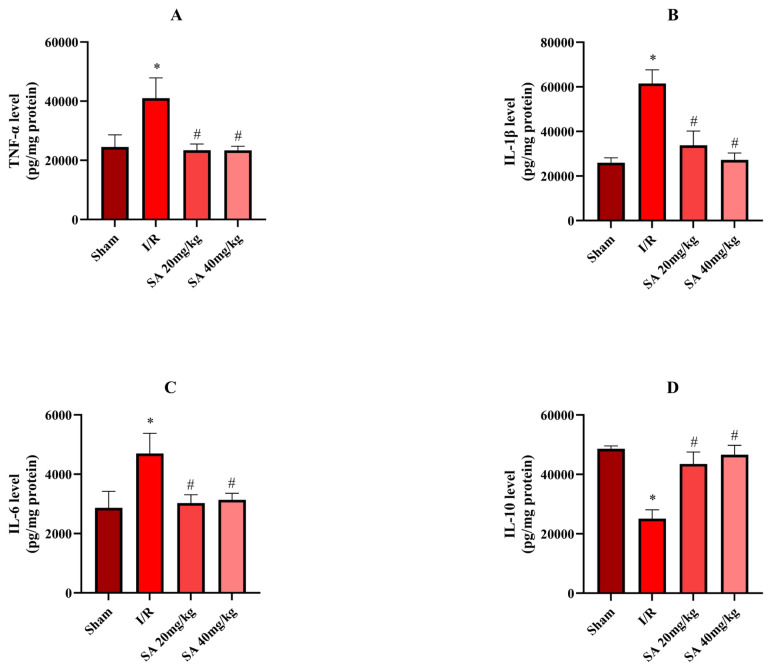
Levels of inflammatory cytokines in kidney tissues across experimental groups. A) TNF-α (pg/mg protein), B) IL-1β (pg/mg protein), C) IL-6 (pg/mg protein), and D) IL-10 (pg/mg protein) levels measured in the sham, I/R, SA 20 mg/kg, and SA 40 mg/kg groups. Data are presented as mean ± standard error of the mean (SEM). *p = 0.018–0.030 compared to the sham group; #p = 0.019–0.027 compared to the I/R group (one-way ANOVA with Tukey’s HSD test).

Inflammatory cytokine levels are depicted in Figure 4. IL-1β, TNF-α, and IL-6 levels were increased in the I/R group compared to the sham group (IL-1β: p = 0.030; TNF-α: p = 0.025; IL-6: p = 0.022; Figures 4A and 4C), whereas IL-10 was decreased (p < 0.029, Figure 4D). SA treatment at 20 mg/kg significantly reduced IL-1β (p = 0.027), TNF-α (p = 0.025), and IL-6 (p = 0.024) levels compared to I/R and elevated IL-10 levels (p = 0.026). The 40 mg/kg dose showed even stronger effects (IL-1β: p = 0.022; TNF-α: p = 0.019; IL-6: p = 0.018; IL-10: p = 0.020). These results confirm the antiinflammatory action of SA in I/R-induced renal injury.

### 3.2. Histopathological examination

**Figure 5:**
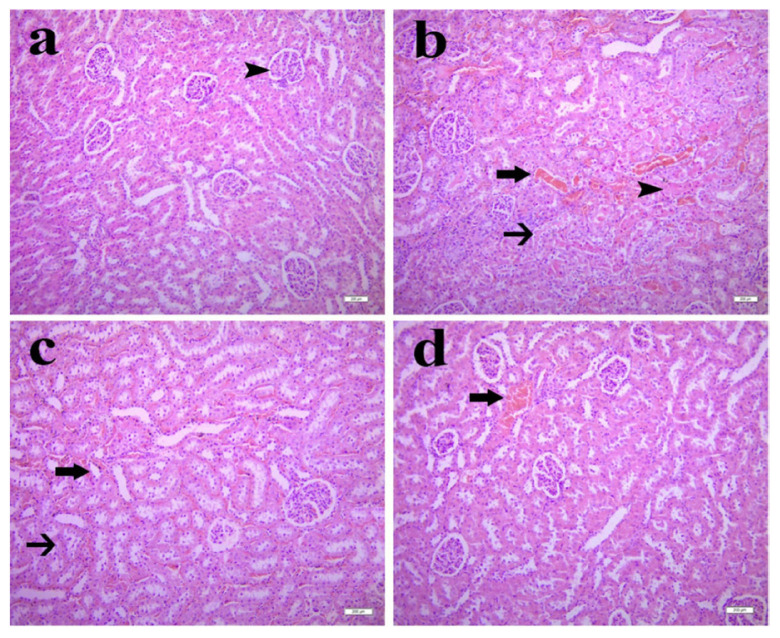
Histopathological evaluation of the effect of SA on I/R-induced kidney injury using H & E staining. A) Sham group showing normal kidney histology (arrowhead = normal glomerulus). B) I/R group showing severe tubular degeneration (thin arrow), necrosis (arrowhead), and hemorrhage (thick arrow). C) SA 20 mg/kg group, mild tubular degeneration (thin arrow) and hemorrhage (thick arrow). D) SA 40 mg/kg group showing mild hemorrhage (thick arrow). H & E staining; 10× magnification.

Figure 5 and Table 1 represent the histological analysis of kidney tissues of the experimental groups. The sham group exhibited normal renal architecture (Figure 5A). In contrast, the I/R group exhibited severe tubular degeneration, necrosis, and interstitial hemorrhage (Figure 5B). SA 20 mg/kg treatment resulted in noticeable mitigation of renal damage, with reduced tubular degeneration, necrosis, and hemorrhage (Figure 5C), while the 40 mg/kg dose further attenuated injury, with only mild changes observed (Figure 5D).

### 3.3. Immunohistochemical examination

**Figure 6:**
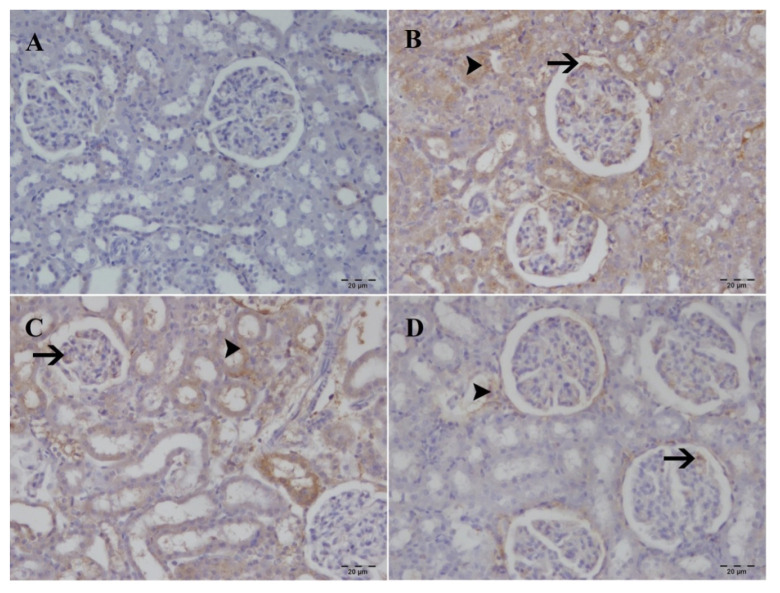
Immunohistochemical analysis of caspase-3 expression in kidney tissues across experimental groups. A) No caspase-3 immunopositivity in the sham group. B) Severe caspase-3 immunopositivity in the tubules (arrowhead) and glomeruli (arrow) of the I/R group. C) Severe caspase-3 immunopositivity observed in the tubules (arrowhead) and glomeruli (arrow) of the SA 20 mg/kg group D) Moderate caspase-3 immunopositivity observed in the tubules (arrowhead) and glomeruli (arrow) of the SA 40 mg/kg group. Immunohistochemical staining; 20× magnification.

**Figure 7:**
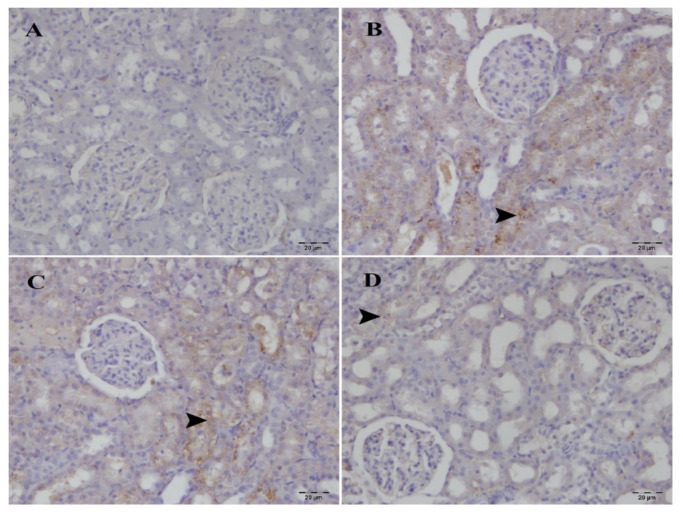
Immunohistochemical analysis of HAVCR1 expression in kidney tissues across experimental groups. A) No HAVCR1 immunopositivity in the sham group. B) Severe HAVCR1 immunopositivity in the tubules (arrowhead) and glomeruli (arrow) of the I/R group. C) Severe HAVCR1 immunopositivity observed in the tubules (arrowhead) and glomeruli (arrow) of the SA 20 mg/kg group. D) Moderate HAVCR1 immunopositivity observed in the tubules (arrowhead) and glomeruli (arrow) of the SA 40 mg/kg group. Immunohistochemical staining; 20× magnification.

Caspase-3 and HAVCR1 expression was not observed in the sham group but was strongly increased in the I/R and SA 20 mg/kg groups. The SA 40 mg/kg group demonstrated moderate immunopositivity (Figures 6 and 7; Table 2). Quantitative analysis (Table 3) confirmed significantly higher caspase-3 and HAVCR1 levels in the I/R and SA 20 mg/kg groups compared to sham (p < 0.05), with no difference between the two. However, the 40 mg/kg SA group showed significantly reduced expression of both markers compared to I/R and SA 20 mg/kg (p < 0.05), indicating dose-dependent protective effects against apoptosis and tubular injury.

## 4. Discussion

This research presents the renoprotective effects of SA in an I/R injury rat model, primarily by the modulation of apoptosis, inflammation, and oxidative stress. Our findings demonstrate that SA administration, particularly at 40 mg/kg, significantly attenuated renal tissue damage, improved antioxidant defense, reduced inflammatory cytokine expression, and suppressed apoptotic signaling, thereby supporting its therapeutic potential in AKI.

Renal I/R injury initiates a multifaceted pathological cascade involving excessive ROS production, inflammatory mediator release, and apoptosis of tubular epithelial cells [33]. In our study, I/R insult resulted in substantial histopathological damage, and these changes were markedly ameliorated by SA treatment, especially at the higher dose—consistent with previous reports on the protective effects of natural polyphenols in kidney injury models [34].

Oxidative stress is a key driver of I/R-induced renal damage, resulting from the imbalance between ROS production and antioxidant defenses [35]. The I/R group exhibited elevated levels of oxidative stress, indicating substantial oxidative burden. SA treatment restored redox homeostasis, corroborating earlier findings of SA’s antioxidative efficacy in nephrotoxic and hepatic injury models [23, 36, 37].

Inflammation plays a central role in AKI progression. Proinflammatory cytokines like IL-6, TNF-α, and IL-1β are involved in leukocyte recruitment, vascular dysfunction, and tubular injury, while IL-10 acts as an antiinflammatory cytokine promoting tissue repair [38, 39]. SA effectively reduced the proinflammatory cytokine levels, reflecting its immunomodulatory capacity. These results align with previous studies demonstrating SA’s ability to modulate inflammatory responses in both renal and neural tissues [40, 41].

Apoptosis is another major contributor to renal I/R injury. Caspase-3 is a crucial executioner caspase involved in epithelial cell apoptosis during I/R, and its overexpression correlates with tissue damage severity [42–44]. In our model, SA significantly downregulated apoptosis, particularly at 40 mg/kg. This observation agrees with earlier research showing SA-mediated suppression of caspase-3 in models of nephrotoxicity and diabetes-related organ injury [45, 46].

HAVCR1 is a well-established biomarker of renal tubular injury and an indicator of epithelial cell stress and regeneration [47]. Our findings revealed that 40 mg/kg SA alleviated HAVCR1 expression, suggesting that SA may protect against tubular injury and facilitate structural recovery. This is consistent with prior studies reporting reduced HAVCR1 expression following administration of phytochemicals with nephroprotective properties [48, 49].

From a translational perspective, SA is a naturally occurring bioactive phenolic compound widely distributed in cereals, fruits, vegetables, and agri-food by-products such as seed coats and bran [50]. Its dietary origin, economic accessibility, and safety profile position it as a promising candidate for development as a nutraceutical or dietary supplement for renal protection. The integration of such compounds into preventive or adjunctive therapeutic strategies could offer a nontoxic, low-cost approach to mitigating renal I/R injury.

Our findings are consistent with studies investigating other polyphenolic compounds, such as resveratrol, quercetin, and curcumin, which have also demonstrated protective effects against renal I/R injury through antioxidant, antiinflammatory, and antiapoptotic mechanisms [51–53]. However, SA is notable for its high abundance in dietary sources and favorable safety profile, underscoring its potential translational value in preventing AKI.

Notably, while prior studies have highlighted the nephroprotective effects of SA in gentamicin- and cisplatin-induced nephrotoxicity, the efficacy of SA in renal I/R injury—a common and clinically relevant cause of AKI—has not been thoroughly investigated. Our findings provide novel insights into the potential of SA in this specific context.

Despite these promising findings, several limitations must be acknowledged. The study was conducted in an acute rat model, and its clinical applicability to human AKI remains to be established. The study employed an acute model of renal I/R injury, and the long-term effects of SA treatment are yet to be determined. Additionally, while SA’s effects on oxidative stress, cytokine levels, and apoptosis were evaluated, the underlying molecular signaling pathways—such as NF-κB, Nrf2, and MAPK—were not explored. This omission limits the translational depth of the study. Future investigations should incorporate molecular assays (e.g., Western blotting, qRT-PCR) to elucidate these pathways, as well as dose-response histological quantification and long-term follow-up assessments, to strengthen the mechanistic understanding and clinical relevance of SA’s protective effects.

In this experimental model of renal I/R injury, SA demonstrated significant renoprotective effects through its antiinflammatory, antioxidant, and antiapoptotic properties. SA treatment, particularly at a dose of 40 mg/kg, effectively restored redox balance, suppressed proinflammatory cytokines, and reduced histopathological and immunohistochemical markers of tubular damage and apoptosis. These findings support the potential of SA as a promising therapeutic candidate for the prevention or attenuation of AKI. Further research, including molecular pathway analyses and clinical investigations, is warranted to explore its translational applicability and long-term safety profile.

## Figures and Tables

**Table 1:** Scoring of histopathological changes for necrosis, hemorrhage, and degeneration in the study groups (None: −, mild: +, moderate: ++, severe: +++).

	Sham	I\R	SA 20 mg/kg	SA 40 mg/kg
**Necrosis**	−	+++	++	+
**Tubular Degeneration**	−	+++	++	+
**Hemorrhage**	−	+++	++	+

I/R: Ischemia-reperfusion; SA: Sinapic acid. Statistical differences among groups were assessed using the Kruskal–Wallis test followed by the Mann–Whitney U test (p < 0.05).

**Table 2:** Scoring of immunohistochemical findings observed in the study groups (None: −, mild: +, moderate: ++, severe: +++).

	Sham	I\R	SA 20 mg/kg	SA 40 mg/kg
**Caspase- 3**	−	+++	+++	++
**HAVCR1**	−	+++	+++	++

I/R: Ischemia-reperfusion; SA: Sinapic acid. Statistical differences among groups were assessed using the Kruskal–Wallis test followed by the Mann–Whitney U test (p < 0.05).

**Table 3:** Statistical analysis of the immunohistochemical findings.

	Sham	I\R	SA 20 mg/kg	SA 40 mg/kg
**Caspase-3**	14.33 ± 2.18^a^	63.24 ± 7.31^c^	64.78 ± 6.68^c^	28.32 ± 7.33^b^
**HAVCR1**	15.48 ± 3.12^a^	65.48 ± 6.92^c^	61.38 ± 4.75^c^	31.22 ± 7.33^b^

I/R: Ischemia-reperfusion; SA: Sinapic acid. Statistical differences among groups were assessed using the Kruskal–Wallis test followed by the Mann–Whitney U test (p < 0.05). Different superscript letters (a, b, c) within a row indicate statistically significant differences between groups (p < 0.05).
